# Formation and Developmental Specification of the Odontogenic and Osteogenic Mesenchymes

**DOI:** 10.3389/fcell.2020.00640

**Published:** 2020-07-17

**Authors:** Eva Svandova, Renata Peterkova, Eva Matalova, Herve Lesot

**Affiliations:** ^1^Laboratory of Odontogenesis and Osteogenesis, Institute of Animal Physiology and Genetics, Academy of Sciences, Brno, Czechia; ^2^Department of Histology and Embryology, Third Faculty of Medicine, Charles University, Prague, Czechia; ^3^Department of Physiology, University of Veterinary and Pharmaceutical Sciences, Brno, Czechia

**Keywords:** development, mouse, mandible, mesenchyme, condensation, odontogenesis, osteogenesis

## Abstract

Within the mandible, the odontogenic and osteogenic mesenchymes develop in a close proximity and form at about the same time. They both originate from the cranial neural crest. These two condensing ecto-mesenchymes are soon separated from each other by a very loose interstitial mesenchyme, whose cells do not express markers suggesting a neural crest origin. The two condensations give rise to mineralized tissues while the loose interstitial mesenchyme, remains as a soft tissue. This is crucial for proper anchorage of mammalian teeth. The situation in all three regions of the mesenchyme was compared with regard to cell heterogeneity. As the development progresses, the early phenotypic differences and the complexity in cell heterogeneity increases. The differences reported here and their evolution during development progressively specifies each of the three compartments. The aim of this review was to discuss the mechanisms underlying condensation in both the odontogenic and osteogenic compartments as well as the progressive differentiation of all three mesenchymes during development. Very early, they show physical and structural differences including cell density, shape and organization as well as the secretion of three distinct matrices, two of which will mineralize. Based on these data, this review highlights the consecutive differences in cell-cell and cell-matrix interactions, which support the cohesion as well as mechanosensing and mechanotransduction. These are involved in the conversion of mechanical energy into biochemical signals, cytoskeletal rearrangements cell differentiation, or collective cell behavior.

## Introduction

During development, epithelially derived organs (e.g., skin appendages, teeth) form from an initial epithelial bud surrounded by a condensed mass of embryonic connective tissue (mesenchyme). The mesenchyme-derived organs (e.g., bones, cartilages) develop from mesenchymal cell condensations (Hall and Miyake, 2000; Giffin et al., 2019).

The odontogenic and osteogenic jaw ecto-mesenchymes originate from cranial neural crest-derived cells (NCDCs). Their condensation takes place in two distinct regions, at about the same time in the embryonic mouse lower jaw. They later diversify into multiple cell types, along with their progressive dental or bone specification (Ruch et al., 1995; Santagati and Rijli, 2003). Cranial neural crest cells are multipotent and differ according to distinct migratory streams and environments at a post-migratory stage (Yuan and Chai, 2019). Both the odontogenic and osteogenic condensations are progressively invaded by exogenous cells, which increases cell diversity. The cellular heterogeneity of the mesenchyme will be discussed below, as well as the mechanisms suggested to be involved in mesenchymal condensation, the progressive specification of the different mesenchymal areas, and the biological interfaces between soft and hard tissues.

The cell diversity in the three mesenchymal areas, including the participation of exogenous cells, is still under investigation in several laboratories. Comparison between situation in all three regions was discussed with regard to their ability to condense and mineralize or not. Particular attention was given to differential cell–cell and cell–matrix interactions and thus mechanosensing and mechanotransduction. All together, the sequential steps and mechanisms involved in it ultimately lead to the formation of a functional unit: a tooth firmly attached to the surrounding mandibular/alveolar bone by means of the non-mineralized periodontal ligament (PDL). Most of the data discussed here are related to the mouse first lower molar, a very common model. However, the general aspects considered here should be applicable to other models.

## Mesenchymal Cell Heterogeneity in the Mandible

**FIGURE 1:**
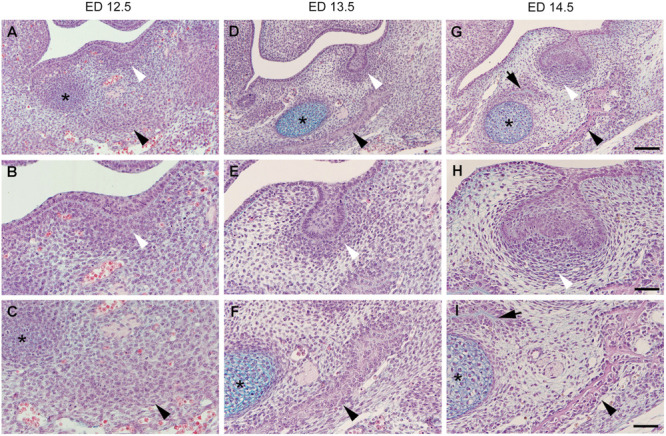
Histological sections of the embryonic mouse lower jaw at the level of M1. The two odontogenic (white arrowhead) and osteogenic (black arrowhead) mesenchymal condensations are distant from each other being separated by a looser interstitial mesenchyme at embryonic day (ED)12.5 **(A–C)**. Jaw bone appears at ED13.5 **(D–F)**, and ED14.5 **(G–I)**, and its development is accelerated on the lateral side (black arrowhead) when compared to the medial side (black arrow). Both the bone and tooth germ grow and approach each other at the expense of the area of the interstitial mesenchyme. The dental follicle cells achieve progressively a typical pattern (compare to Figure 2). Although mineralization will take place in both osteogenic and odontogenic condensations, the interstitial mesenchyme (see Figure 2) will never mineralize in normal conditions. * Refers to the Meckel’s cartilage, Bars = 100 mm **(A,D,G)** and 50 mm **(B,C,E,F,H,I)**.

In the mouse mandible at embryonic day (ED)12.5, two distant condensations are separated from each other by a looser uncondensed mesenchyme: an osteogenic one, deep in the embryonic jaw and the odontogenic one, adjacent to the thickened dental epithelium/dental lamina in the prospective first molar (M1) region (Figures 1A–C). At early stages, the external limits of the two condensations can hardly be determined since there is a continuous decreasing gradient in cell density toward the surrounding uncondensed mesenchyme (Figures 1A,B). Both the odontogenic mesenchymal condensation and the dental epithelium represent the ecto-mesenchymal and epithelial components of a developing tooth primordium, respectively.

At ED13.5, the odontogenic condensation becomes more pronounced and surrounds the dental epithelium, which acquires the shape of a tooth bud (Figures 1D,E). A bone matrix is already visible in the osteogenic condensation in the mouse lower jaw (Figure 1F). At early stages, the intramembranous bone formation is characterized by apposition only. The characteristic of intramembranous bone formation is the absence of a chondrogenic step as it exists during endochondral bone development. Progenitor cells present in the condensation differentiate directly into osteoblasts (Berendsen and Olsen, 2015). Signaling and transcriptional regulation, involved in osteoblasts differentiation, have been investigated (for review, see Huang et al., 2007; Ferguson and Atit, 2019).

Within 2 days, from ED13 to ED15, when cell differentiation occurs, the osteogenic condensation turns into a vascularized bone containing bone specific cells: (e.g., Alfaqeeh et al., 2013; Vesela et al., 2019). From ED15, the bony crypt around the dental primordia starts to be remodeled, mostly by bone resorption (Radlanski et al., 2015).

**FIGURE 2:**
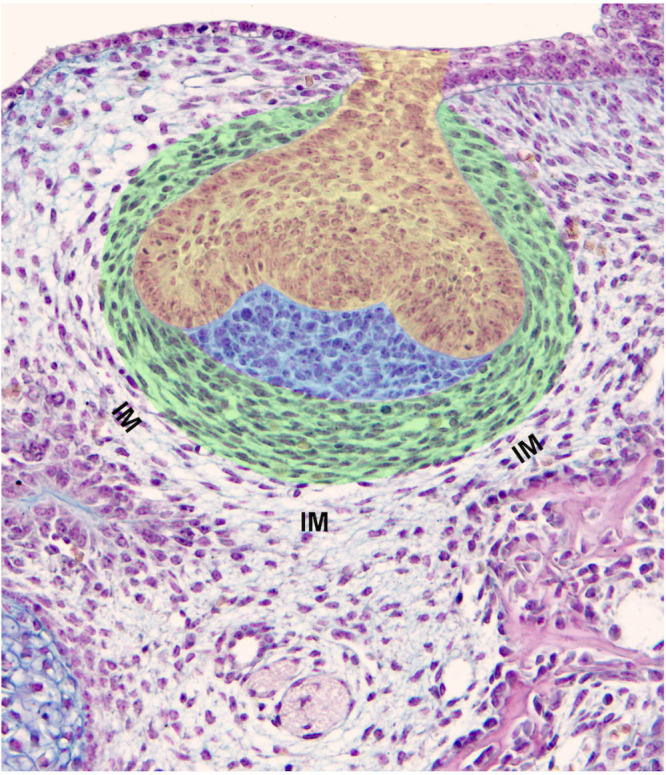
Components of a tooth germ and surrounding tissues. Mouse first lower molar germ at ED14.5 exhibits a clear subdivision of dental mesenchyme including the dental papilla (blue) and dental follicle/dental sac (green). Cells of the dental papilla show neither specific shape nor spatial organization. Conversely, cells of the dental sac show an elongated shape being arranged in concentric rings (green). The contemporaneous presence of increased cell density, their specific shape and pattern are criteria for the determination of the dental sac boundary. The dental epithelium is at a cap stage (yellow). IM, interstitial mesenchyme.

The transition from the epithelial tooth bud to cap is accompanied by the differentiation of the former odontogenic mesenchymal condensation. This condensation gives rise to two compartments: (1) the dental papilla encapsulated by the epithelial cap, and (2) the dental follicle (or dental sac), which surrounds the epithelial cap and papilla (Figure 2). The papilla cells are progressively specified by sequential epithelial-mesenchymal interactions, and the preodontoblasts/odontoblasts lineage will later be controlled by the inner dental epithelium (Mina and Kollar, 1987; Lumsden, 1988; Ruch et al., 1995). The cell heterogeneity in the dental papilla becomes more and more complex (Keller et al., 2012; Kokten et al., 2014b; Krivanek et al., 2017). The characteristic shape and pattern of cells within the dental follicle allows its delineation from the surrounding loose interstitial mesenchyme (Figures 1H, 2).

At ED14.5, dispersed cells of the interstitial mesenchyme are interposed between the external cells of the dental follicle and the osteogenic condensation (Figures 1G–I, 2). Very quickly, this mesenchyme shows further peculiarity. Immunostainings for VEGFR2, CD31, CD34 showed that the interstitial mesenchyme is vascularized at ED14, slightly before the dental mesenchyme and before the forming bone itself (Nait Lechguer et al., 2008; Keller et al., 2012; Vesela et al., 2019).

This interstitial mesenchyme is maintained at ED15, when the tooth germ has reached the late cap stage and bone has developed. How far these cells will later participate to the PDL and possibly even to the osteogenic mesenchyme is unknown since cell tracing has not been performed for this population (Zhao et al., 2013), in opposition to cranial neural crest cells (Chai et al., 2000), or mesenchymal cells facing the tip of the tooth bud (Rothova et al., 2012). Dissociation-reassociation experiments were performed using dissociated papilla and follicle cells from cap stage (ED14) molar germs (see Figure 1 in Kokten et al., 2014a). When re-associated with an intact enamel organ or dissociated epithelial cells, it was possible to reconstitute a whole tooth. This showed that although all initial positional information had been lost, mesenchymal cells at this stage can reconstitute a whole tooth germ. This demonstrated a high cell plasticity (Hu et al., 2005; Kuchler-Bopp et al., 2011), which decreases at later stages (Keller et al., 2011). This was in agreement with labeling and fate-mapping experiments showing that cells from the odontogenic condensation at the bud stage could participate in the follicle at the cap stage (Rothova et al., 2012). These experiments still raise the question of understanding why do these cells migrate, when cells of the follicle are already present there at that stage (Figure 2). One possibility is that the migrating dental mesenchymal cells increase the heterogeneity of the dental follicle and bring new necessary cell type(s) still absent before. It would thus be of interest to investigate in more details the heterogeneity of dental follicle cells before and after cell migration. Two other questions to be addressed concern: (1) the disruption of cell-cell/cell-matrix interactions within the dental papilla to allow cell migration and (2) the nature of the signals driving such a long distance migration and mediating cell targeting.

Cells from the interstitial mesenchyme (Figure 2), or at least a sub-population, are particularly important as possibly contributing to the development of the future PDL. At early developmental stage, and in opposition to the dental mesenchyme and follicle cells, interstitial mesenchymal cells do not express markers for NCDCs (Chai et al., 2000). It has been proposed that gingival connective tissue fibroblasts originate from perifollicular mesenchyme, a derivative of the stomodeal mesoderm (Cho and Garant, 2000). This particular origin of least part of the PDL fibroblasts might explain their specific properties and differences with the neighboring ecto-mesenchymal odontogenic and osteogenic condensations. Among these peculiarities, interstitial mesenchymal cells produce an extracellular matrix (ECM) that will not mineralize, thus impairing tooth ankylosis during later development.

## Mechanisms Involved in Mesenchymal Cell Condensations

**SCHEME 1:**
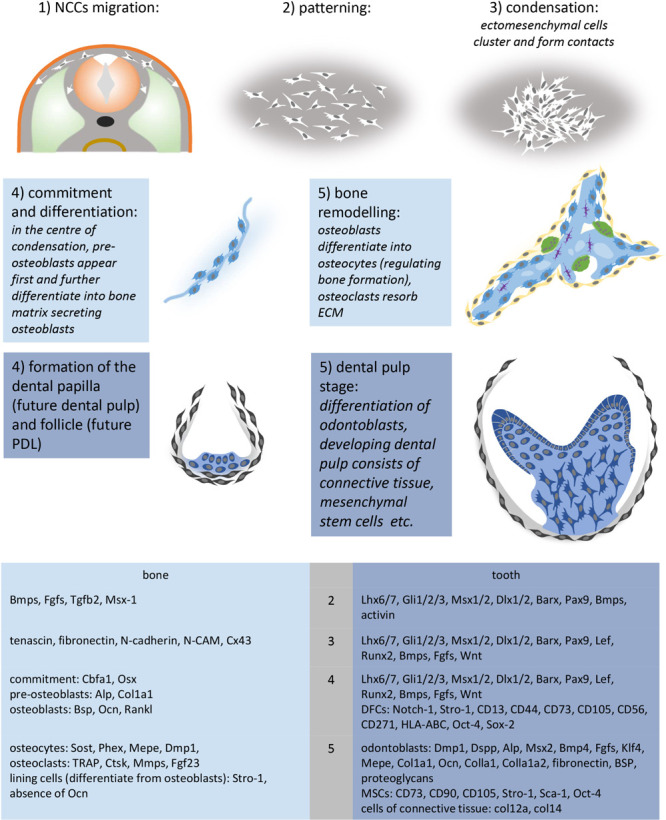
Crucial steps and molecules involved in the development of mesenchymal condensations. Molecular markers were summarized according to Casagrande et al. (2010), Dallas and Bonewald (2010), Everts et al. (2002), Ferguson and Atit (2019), Franz-Odendaal (2011), Giffin et al. (2019), Hall and Miyake (2000), Ledesma-Martínez et al. (2016), Lin et al., 2011, 2013; Nanci et al. (2008), Zhou et al. (2019), and Zvackova et al. (2017). Ectoderm (orange), NCCs (white), somite (light green), osteoblasts (light blue), lining cells – osteogenic cells (yellow), osteocytes (purple), osteoclasts (green), dental follicle (gray), dental papilla (dark blue), odontoblasts (dark blue-columnar).

Cell condensation is a fundamental mechanism involved in morphogenesis. Condensations of mesenchymal cells take place during the development of almost all tissues and organs, including skin appendages, primordia of sensory organs, parenchymatous organs and musculo-skeletal structures (for review, see Giffin et al., 2019). Cell condensation occurs when a population of originally dispersed cells aggregates to differentiate into a specific cell/tissue type (Scheme 1; Hall and Miyake, 2000). The condensation results from altered mitotic activity, absence of centrifugal cell movement and/or centripetal cell aggregation (Hall and Miyake, 1995). According to Shyer et al. (2017), mesenchymal cells have an intrinsic potential to aggregate. The increase in cell density might facilitate cell–cell communication, possibly required for subsequent lineage acquisition (McKeown et al., 2005). However, the odontogenic and osteogenic mesenchymal condensations are separated by a non-condensed region. This illustrates early differences in the characteristics of mesenchymal cells, despite they are very next to each other. Each of these three cases reflect collective cell behavior, where mechanobiology might play an important role (Ladoux and Mège, 2017). The mesenchymal cell condensation, as it appears in early stage of odontogenesis and mandibular osteogenesis, precedes blood vessels ingrowth. This might be a general situation to be considered for regenerative medicine (Takebe et al., 2015). Furthermore, during tooth development, vascularization is anticipated in the interstitial mesenchyme, and blood vessels do not enter the condensed mesenchyme before the cap stage tooth germ as stated above. Similarly, blood vessels do not enter the osteogenic condensation before ED14 (Vesela et al., 2019).

### Odontogenic Condensation

Condensation of the dental mesenchyme occurs during early steps of odontogenesis. Changes in the cell shape or size were suggested to possibly mediate this phenomenon (Mammoto et al., 2011, 2013). At this stage, histology did not confirm it, but showed change in cell density (Figures 1B,E). A decrease in intercellular spaces was obvious, which could be related to a change in either the ECM itself, in cell–cell interactions, or both. At the bud stage, syndecan and tenascin were detected within the odontogenic mesenchymal condensation and might be involved the in condensation process (Vainio and Thesleff, 1992; Thesleff et al., 1996). Collagen type VI, also present there, has been suggested to play a key role by stabilizing odontogenic cell condensation (Mammoto et al., 2015). According to these authors, cross-linking of collagen type VI by lysyl-oxidase would be involved and interfering with it would change the size of the condensation.

The condensation of the odontogenic mesenchyme appears to be regulated by signaling from the bud epithelium (Dassule and McMahon, 1998). Little is known about this signaling. Mammoto et al. (2011) showed that during mouse tooth development, FGF8 and (semaphorin) SEMA3F, produced by early dental epithelium, were causally involved in mesenchymal cell condensation. This mechanical compaction induced changes in the expression of transcription factors (PAX9, MSX1) and a growth factor (BMP4). These authors concluded that mechanical compression of the mesenchyme is necessary to induce tooth-specific cell fate although a condensation also takes place before osteogenic markers are expressed. The fact that one is in contact with an epithelium, while the other is not, is probably much more important. This has been very precisely documented in the developing tooth at later stage. In case of odontoblasts layer versus sub-odontoblastic cells, this is determined after the last cell division. Among the two daughter cells, the one in contact with a stage-specific basement membrane will differentiate as an odontoblast and elongate, while the other one will gets a flatten shape (Ruch et al., 1982, 1995; Ruch, 1998). However, *in vitro* experiments showed that growth factors, such as TGFbeta-1 or -3, when immobilized by heparin, can replace the inner dental epithelium and basement membrane, and induce preodontoblasts to odontoblasts differentiation (Ruch et al., 1995). BMP-2, -4, and -7 can induce it as well, although in more restricted areas. This involves a change in cell shape, elongation, as well as cytological and functional differentiation (polarized secretion of predentin), as it occurs in physiological conditions (Ruch, 1998).

In case of skin and follicle patterning in avian, Shyer et al. (2017) suggested an inverse mechanism, where mesenchymal cell condensation would change the organization of adjacent epidermal cells by triggering a mechanosensitive activation of beta-catenin in these cells.

### Osteogenic Condensation

During development, a pre-osteogenic phase characterized by mesenchymal cell condensation (for review, see Hall and Miyake, 2000; Jabalee et al., 2013) is followed by post-condensation stage, including osteogenic cell differentiation, vascularization, innervation and mineralization of the ECM. However, these pre- and post-condensation stages remain connected (Dunlop and Hall, 1995). Although the mechanism mediating it remains unclear, mesenchymal cell condensation represents the initial stage of osteogenic specification. Hall and Miyake (2000) have proposed a series of molecules possibly involved in it (Scheme 1).

Fibroblast growth factors (FGFs) as well as bone morphogenetic proteins (BMPs) have been suggested to play a role in the initiation of condensation (Wu et al., 2007; Javed et al., 2010). Tissue cultures and recombinations *in vitro* showed that mandibular osteogenesis involves post-migratory neural crest-derived ecto-mesenchymal cells and their interaction with a mandibular epithelium (Hall, 1980; MacDonald and Hall, 2001). These experimental results raise interesting questions about the signaling process, given the long distance between the initially condensing osteogenic mesenchyme and epithelial tissues *in situ.*

The process of condensation itself requires changes in the local tissue mechanical properties (Mammoto et al., 2011). The generation of contractile forces within mesenchymal cells has been suspected (Oster et al., 1983; Ray and Chapman, 2015). Most of the data to support it and their role in mechanotransduction during osteogenesis have been obtained from *in vitro* experiments (Discher et al., 2005; Komatsu et al., 2018; Giffin et al., 2019). Concerning cell–cell interactions, three connexins, involved in the formation of gap junctions have been investigated during initial mandibular osteogenesis in the chick embryo. Among these, connexin43 (Cx43) appeared to be associated with the condensation of mesenchyme and the earliest stages of osteogenesis (Minkoff et al., 1994). Cx43 gene deletion in the mouse also leads to cell autonomous osteoblast dysfunction and delayed mineralization (Lecanda et al., 2000). Cx43-containing gap junctions thus seem to play an important role in the intercellular communication between the interconnected bone cells network (osteoprogenitor cells, osteoblast, and osteocytes) (Moorer et al., 2017). Similarly, the possible role of cadherins, which mediate interactions with the cytoskeleton of adjacent cells, has been investigated *in vitro* (Griffin et al., 2017). Adhesion junctions are mechanosensing structures, which play diverse roles. Among these, they regulate the expression of transcription factors during osteogenesis (Guntur et al., 2012). It would thus be of interest to investigate the pattern of cadherins during early mandibular/alveolar bone development and to compare it with the situation in neighbor interstitial mesenchyme.

## Mesenchymes Specification and Differentiation During Development

**SCHEME 2:**
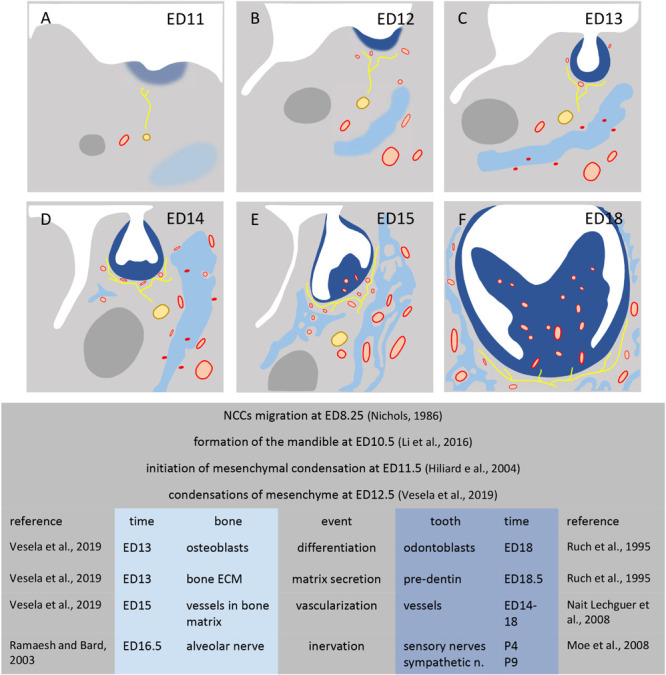
Development of mesenchymal condensations in mouse mandible. Figures show the mandible at stage ED11 **(A)**, ED12 **(B)**, ED13 **(C)**, ED14 **(D)**, ED15 **(E)**, ED18 **(F)**. Dental condensation (dark blue), bone condensation (light blue), Meckel’s cartilage (dark gray), nerves (yellow), vessels (red).

All three mesenchymes (odontogenous, osteogenous, and intermediary) progressively consist in heterogeneous cell types, different in each case and changing during development. In all three cases, besides the presence/absence of NCDCs, exogenous cells take part in cell heterogeneity. Most of these exogenous cells enter together during vascularization, and later during innervation (Scheme 2).

### Dental Mesenchyme

The interactions of the odontogenic neural crest-derived mesenchymal cells with the oral epithelium determine a specific fate, very different from that of the other set of condensing osteogenic mesenchymal cells.

The dental papilla ecto-mesenchyme is clearly defined from the cap stage as part of condensed mesenchyme surrounded by the dental epithelium up to cervical loop, while the rest of the odontogenous mesenchyme gives rise to the dental follicle (Figure 2). Several successive steps lead to the histo-differentiation of the dental papilla and follicle mesenchymes, which are controlled by time- and space-specific epithelial-mesenchymal interactions (MacNeil and Thomas, 1993; Ruch et al., 1995; Thesleff et al., 1995; Miletich and Sharpe, 2003). The first odontoblasts differentiate at ED18. At post-natal day (PN) 1, they become positive for CD73, a 5′nucleotidase, which might be related to their functional differentiation. This 5′nucleotidase as well as alkaline phosphatase use AMP as a substrate (Andrade et al., 2008; Yegutkin, 2008) and could play a role in the expression of osteocalcin, which takes place in mature odontoblasts (Bidder et al., 1998). Odontoblasts were transiently positive for CD90 at PN1 and negative again at PN4 (Keller et al., 2012).

In opposition to clearly distinct cell compartments in the enamel organ, histology does not show anything similar in the forming dental papilla, except for preodontoblast/odontoblast, sub-odontoblastic layers and blood vessels.

Immunostaining revealed the cellular heterogeneity within the dental mesenchyme and implantation experiments in GFP mice were necessary to distinguish the endogenous/exogenous origin of these cells (Kuchler-Bopp et al., 2011; Keller et al., 2012; Kokten et al., 2014b). In the first lower molar, the timing of mesenchymal specification by the epithelium varies along the antero-posterior axis of the tooth and also along the buccal-lingual one (Nakatomi et al., 2013). In both cases, these local variations in the mesenchyme follow the pattern of cusps formation and, within one cusp, are controlled by the specific geometric determination in the inner dental epithelium (Obara and Lesot, 2007; Nakatomi et al., 2013).

Immunostaining for cell surface markers illustrated the cell heterogeneity with distinct patterns in the dental mesenchyme (Keller et al., 2012). The different cell populations also showed a differential timing in their appearance. Amongst these cells some are external to the dental papilla. At the cap stage, the first blood vessels penetrate the dental mesenchyme (Nait Lechguer et al., 2008) and their mesodermal origin has been reported (Rothova et al., 2011). When cultured tooth germs or dental cell–cells re-associations were implanted under the skin in GFP mice, both were re-vascularized. GFP-positive cells from the host were associated with blood vessels (i.e., cells expressing CD34, CD146, and later alpha-SMA) (Nait Lechguer et al., 2008). Complementary experiments showed that CD90-positive cells, and blood vessels associated Sca-1-positive cells can be either GFP-positive or negative, indicating that, in both cases, they still consist of heterogeneous populations, at least regarding their origins (Keller et al., 2012). The immunodetection of CD31, 34, and 146 showed that at ED14, while blood vessels were already present in the interstitial mesenchyme, they only started to enter the dental follicle. They were detected in the whole papilla mesenchyme at ED18 up to cells underlying the odontoblasts layer (Keller et al., 2012). Microvessels reaching the odontoblast layer at PN7 were detected after staining for CD34, collagen type IV or CD146 (Kokten et al., 2014b). Multiple double stainings showed that all three antigens were co-localized. These capillaries might be involved in the transport of calcium necessary for dentin mineralization (Bishop, 1987).

At ED18 also, CD45-positive cells were detected in the dental papilla and follicle, while CD90-positive cells were restricted to the papilla. Several dendritic cell types resident or not were present in the dental mesenchyme (Zhang et al., 2006; Keller et al., 2012). They included immunocompetent cells (Okiji et al., 1992, 1997; Kaneko et al., 2008), which can be recruited to participate in reparative processes (Jontell et al., 1998; Farges et al., 2003; Goldberg et al., 2008). From PN1, Sca-1 was detected in the dental pulp but still, very few cells were visualized. At PN4, this antigen was mainly expressed by cells associated to the largest blood vessels at the apical part of the papilla as also observed for alpha-SMA (Keller et al., 2012).

Sensory nerves enter the dental mesenchyme at PN3-4, while sympathetic nerves involved in vasoregulation penetrate this tissue much later, at PN9 (Tabata et al., 1998; Moe et al., 2008). Sensory and sympathetic axons can make contacts with dental pulp vessels, but most of them are sensory ones (Byers, 1984; Moe et al., 2008). In the odontoblast layer at PN7, axons were very frequently detected in close proximity with capillaries as seen after double staining for peripherin and either CD34 or collagen IV, or CD146 and confirmed by transmission electron microscopy (Kokten et al., 2014b). At PN7, when reached by axons, odontoblasts could function as mechanosensory cells (Allard et al., 2006; Magloire et al., 2009, 2010). Using confocal laser scanning microscopy, Farahani et al. (2012) showed that the heterogeneity of odontoblasts-associated cells increased precisely at that time.

Double staining for S100-beta and GFAP, two markers for glial cells, did not completely overlap, showing the existence of distinct glial cell types. S100-beta positive cells also showed more proximity with microvessels than GFAP-positive ones. Glial cells reached odontoblasts at PN7 (Kokten et al., 2014b). Schwann cells were present near the basal pole of odontoblasts from PN9, but not detected in the odontoblast layer before PN60 (Corpron and Avery, 1973).

Several different stem cell populations have been identified in the papilla and dental follicle, which also contribute to the cellular heterogeneity of these tissues (Shi et al., 2005; Yen and Sharpe, 2008; Yu et al., 2015; Sharpe, 2016). Genetic lineage tracing was performed to better understand the origin and properties of mesenchymal stem cells (MSCs) *in vivo* (Sharpe, 2016). When tested after transplantation, they were reported to exhibit different phenotypic and functional properties (for review, see Volponi and Sharpe, 2013).

Altogether, these results exemplify very dynamic changes in the heterogeneity of dental mesenchymal cells during odontogenesis. This illustrates a further progressive specification of papilla cells and the contribution of exogenous cells entering this mesenchyme from about ED14 (Moe et al., 2008; Keller et al., 2012; Krivanek et al., 2017), after it became vascularized (Chai et al., 2000; Nait Lechguer et al., 2008; Rothova et al., 2011; Feng et al., 2011).

### Mandibular Bone Specification

Amongst bone cells, osteocalcin-positive osteoblasts are detected in the osteogenic condensation already at ED13 in mouse embryos. Osteoblasts directly differentiate from condensed ecto-mesenchymal cells and give rise to osteocytes. Parathormone (PTH) shares its membrane-bound parathyroid hormone 1 receptor (PTH1r) with parathyroid hormone-related protein (PTHrP). PTH1r and PTHrP are involved in osteoblast maturation in the mandibular/alveolar bone by stimulating osteocalcin expression (Martin, 2005; Bobek et al., 2020). At ED15, these osteocytes are PTH1r- and VDR-positive. Glucocorticoids are involved in the control of osteocyte differentiation (Alemi et al., 2018). Osteocytes, as forming a canalicular network, are thought to constitute a mechanosensory network, involved in mechanotransduction (Burger and Klein-Nulend, 1999; Bonewald and Johnson, 2008). LRP5, a co-receptor of Wnt, is essential for mechanotransduction, a mechanism by which cells convert mechanical energy into biochemical signals (Sawakami et al., 2006). Alveolar bone is a highly mechanoresponsive tissue. Primary cilia act as mechano- and chemosensors, transferring signals from the extracellular to intracellular compartments. Cilia also modulate the non-canonical Wnt signaling (Yavropoulou and Yovos, 2016).

Osteoclasts, responsible for bone resorption have precursors in the bone marrow. The migration of these precursors is thus associated to the vascularization. TRAP-positive mononucleated cells were detected when blood vessels start to penetrate the osteogenic condensation, at ED14 (Bobek et al., 2020). TRAP-positive osteoclasts are detected at ED14, and multinucleated osteoclasts at E15 (Bobek et al., 2020). Protocadherin-7 is involved in osteoclastogenesis by promoting cell-cell fusion (Nakamura et al., 2014). The activation of osteoclasts is mediated by RANKL produced by PTH-stimulated osteoblasts (Boyce and Xing, 2007). RANKL-RANK-OPG is an essential signaling network for osteoblast-osteoclast cross-talk (Nakagawa et al., 1998).

Besides bone cells *stricto sensu*, blood vessels penetrate the osteogenous condensation at ED14, and the bone structure is vascularized by ED15 (Vesela et al., 2019). As reported in the case of the dental papilla, blood vessels play a major role in bone homeostasis, in regulating the osteogenic microenvironment, bringing minerals, growth factors, and osteogenic progenitor cells (e.g., Grosso et al., 2017). Endothelial cells secrete osteogenic factors, such as BMP-2 and BMP-4, which support osteoblast differentiation. VEGF regulates in a dose dependent way the expression SEMA3A in endothelial cells. SEMA3A inhibits osteoclasts differentiation, thus stimulating bone development (Hayashi et al., 2012; Kenan et al., 2019). Conversely, the deletion of Vegf in Osterix-positive osteoblast precursors reduces mandibular ossification (Wang et al., 2007; Hill et al., 2015).

In human, the alveolar nerve issued from the trigeminal ganglion extends in the lower jaw before bone tissue forms and early stages of bone formation take place in close relationship with the mandibular nerve (Kjaer, 1990). Interactions between neurons and bone tissue have been documented in several models during skeletal development and repair. However, only scarce data are available about the innervation of the developing mandibular/alveolar bone. During development, CGRP (calcitonin gene-related peptide) is required for the formation of mandibular bone. When associated with blood vessels, it controls local blood flow (Hill and Elde, 1991). ISH showed that at ED14.5, Cgrp is expressed by mesenchymal cells surrounding the bone matrix and blood vessels (Maeda et al., 2017). Nerves, bone cells, and immune cells, influence bone homeostasis through a local secretion SEMA3A, which is involved in the regulation of osteoblastogenesis and osteoclastogenesis (Jabalee et al., 2013; Xu, 2014).

Schwann cells can play an active role in osteogenesis in the mandibular bone, but all studies were performed in case of distraction osteogenesis, a surgical separation of the jaw bone to elicit new bone growth in the gap (Farhadieh et al., 2003; Byun et al., 2008). It would thus be of interest to investigate the innervation during mandibular/alveolar bone formation in the mouse embryo, and compare what happens during distraction osteogenesis with what can be observed during development. A mouse model has been set up to investigate the nerve dependency of the mouse skeletal stem cell (mSSC), progenitor cells responsible for skeletal development and repair (Jones et al., 2019). According to these authors, the mandibular bone repair is compromised after inferior alveolar nerve disruption and denervation, as a result of functional deficiencies of mSSCs. A partial rescue of the denervated phenotype has been observed after Schwann cell transplantation and by Schwann cell-derived growth factors, suggesting that mSSCs would be dependent on paracrine factors secreted by Schwann cells (Jones et al., 2019).

Jaw bones contain very limited quantities of orofacial bone/bone-marrow-derived MSCs. Their isolation thus requires a specific methodology. When compared to long bone marrow-derived MSCs, orofacial bone/bone-marrow-derived MSCs demonstrated specific immunomodulatory properties and different capacities *in vitro*, thus appearing as a unique population of MSCs (Yamaza et al., 2011). *In vitro* experiments showed that Wnt/β-catenin signaling pathway is involved in the regulation of MSCs migration (He et al., 2018). *In vitro* experiments showed that the chemical and physical properties of the microenvironment modulate the commitment and fate of mesenchymal stromal/stem cells (MSSCs). Intermittent fluid shear stress, a potent and physiologically relevant mechanical stimulus, regulates osteogenic differentiation of MSCs through a transient receptor potential melastatin 7 (TRPM7). TRPM7 is a member of a family of TRP mechanosensitive cation channels with a cytosolic α-kinase domain that interferes with cytoskeletal rearrangements (Clark et al., 2006; Liu et al., 2015). TRP responds to intracellular and extracellular stimuli. Liu et al. (2015) suggested that TRPM7 has differential roles in endochondral and intramembranous ossification.

### From the Dental Follicle to the Periodontal Ligament

Although the dental follicle and dental papilla have the same ecto-mesenchymal origin (Ten Cate, 1997), they soon diverge, showing distinct cell shape and arrangement (Figure 2). Cells initially located in the interstitial mesenchyme (Figure 2) participate in the formation of the PDL. Progenitor cells present in the dental follicle then give rise to cementoblasts, and possibly contribute to the alveolar bone osteoblasts (Diekwisch, 2002 and references therein; Diep et al., 2009; Dangaria et al., 2011).

### Progressive Cell Heterogeneity in the Interstitial Mesenchyme

As stated above, cells from the odontogenic condensation at the bud stage later form the dental papilla in the epithelial cap. Some have been suggested to migrate and participate in the follicle at the cap and bell stages (Osborn and Price, 1988; Rothova et al., 2012). The interstitial mesenchyme is highly vascularized already at ED14 (Nait Lechguer et al., 2008; Keller et al., 2012). It also starts to be innervated at the cap stage (Scheme 2; Luukko and Kettunen, 2014). This very complex heterogeneous cell population is progressively set up from ED14. At the same time and later, several distinct stem cells have been described, which play complementary roles (Luan et al., 2006). SEMA3A has been detected in the dental follicle from ED15 (Wada et al., 2014). These authors suggested that, besides its important role in innervation (Moe et al., 2012; Luukko and Kettunen, 2014), SEMA3A might also induce the conversion of PDL cells into mesenchymal-stem-like cells (Wada et al., 2014; Tomokiyo et al., 2019).

### Periodontal Ligament

**FIGURE 3:**
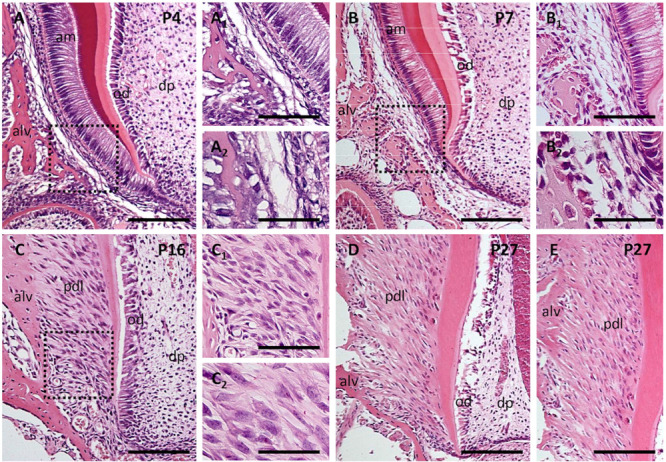
Postnatal development of periodontal ligaments at P4 **(A,A_1_,A_2_)**, P7 **(B,B_1_,B_2_)**, P16 **(C,C_1_,C_2_)**, and P27 **(D,E)**. Sections were stained by hematoxylin-eosin. Alv, alveolar bone; am, ameloblasts; dp, dental pulp; od, odontoblasts; pdl, periodontal ligament. The scale bar represents: 100 μm (in figures **A–E**); 50 μm (in figures **A1,B1,C1**); 25 μm (in figures **A2,B2,C2**).

The PDL forms and differentiates quite late postnatally. At PN4 (Figures 3A, A1, A2). Even at PN7 (Figures 3B, B1, B2), the developing PDL does not yet show any characteristic organization (Keller et al., 2015, Figure 28.3a).

The PDL consists of heterogeneous cell populations (e.g., Fleischmannova et al., 2010). These include fibroblasts (Lekic and McCulloch, 1996), vascular endothelial cells (Saghiri et al., 2018) and associated pericytes (Roguljic et al., 2013), macrophages (Epelman et al., 2014) and dendritic cells, acting as immunocompetent cells (Kaneko et al., 2008). Using the rat as a model, Kaneko et al. (2008) observed that the molecular phenotype of resident dendritic cells is heterogeneous, probably reflecting differences in their maturation/activation. From PN7 (Figures 3B, B1) to PN16 (Figures 3C, C1, C2) there is a striking increase in the density of PDL cells. A rapid growth of the PDL was reported to take place in between PN12 and PN16 (Lungova et al., 2011).

The ECM of the PDL consists in collagens, non-collagenous proteins and glycoproteins, enzymes such as MMPs and growth factors, such as FGFs, BMPs, platelet-derived growth factors (PDGFs). *In vitro* experiments showed that FGF2 inhibits the mineralization of the PDL matrix (Dangaria et al., 2009). The main role of the PDL is the anchoring of the tooth to the surrounding mandibular/alveolar bone and providing a space to allow tooth movement within the bone socket. This is mediated by bundles of collagen fibers ending as Sharpey’s fibers embedded in the cementum on one side and bone on the other (Figures 3D,E; Ho et al., 2007; Lee et al., 2015; Hirashima et al., 2020a). Based on immunoelectron microscopy, Huang et al. (1991) have proposed that periodontal and Sharpey’s fibers consist in co-fibrils of type I and type III collagens, which is supported by the similar flexibility of alfa-1 chains in both collagen types and their possible involvement in elastic energy storage during stretching (Silver et al., 2002). The PDL matrix also includes collagens types V, VI, and XII, as well as type XIV at late stage (Beertsen et al., 1997; Zvackova et al., 2017). Fibril-associated collagens with interrupted triple helices (FACITs) such as collagens XII and XIV are also involved in the regulation of collagen fibrillogenesis (Ricard-Blum, 2011). Transgenic mice carrying a mutation on collagen XII exhibit a disorganized arrangement of collagen fibers in the PDL (Reichenberger et al., 2000). Analyzing double KO mice, Wang et al. (2014) stressed the importance of small leucine-rich proteoglycans (SLRPs) in the maintenance of periodontal homeostasis, which involves the regulation of TGFβ/BMP signaling, matrix turnover, and collagen organization (Matheson et al., 2005; Wang et al., 2014; Zvackova et al., 2017).

The periodontal tissues are highly vascularized (Suphasiriroj et al., 2013; Lee et al., 2015) and close associations between blood vessels and sympathetic nerves allow their mediating vasoregulatory activities (Luukko and Kettunen, 2016). From PN4, immunostaining for peripherin showed the presence of axons in the PDL (Kokten et al., 2014a). Glial and Schwann cells are present in the PDL (Yamamoto et al., 2012; Kokten et al., 2014a). Phenotypic differences were also found when comparing Schwann cells in the pulp and PDL, based on their reactivity to antibodies to GFAP (Byers et al., 2004).

The PDL is exposed to various mechanical stresses associated with mastication. This leads to an active tissue remodeling, which involves progenitor cells (Hirashima et al., 2020a). Mechanical stimuli are important to orient the fate of stem cells. PDL stem cells (PDLSCs) can directly respond to mechanical forces. Both the ECM and mechanical forces can modulate the ability of PDLCs to sustain tissue development and maintenance (Nava et al., 2012; Sun et al., 2012). Heterogeneous populations of stem/progenitor cells have been identified there (Luan et al., 2006; Shinagawa-Ohama et al., 2017; Tomokiyo et al., 2018; Trubiani et al., 2019). PDLSCs showed osteogenic (Yang et al., 2017), cementogenic (Seo et al., 2004; Gauthier et al., 2017) and immunomodulatory properties (Wada et al., 2009). PDLSCs can also stimulate angiogenesis (Yeasmin et al., 2014; Amorim et al., 2016; Bae et al., 2017) or engage in neurogenesis (Ng et al., 2019). In case of angiogenesis, it seems a subset of PDLSCs (CD105+) may play a pivotal role, which would involve neuropilin-2 (NRP-2) (Amorim et al., 2016). PDLSCs can also contribute to the repair and regeneration of the periodontium (Tomokiyo et al., 2019).

## Biological Interfaces: Spatial Limits for Mineralization

There are spatial limits for mineralizing areas in the tooth (predentin/dentin) as well as in the cementum/PDL and PDL/bone junctions (McKee and Nanci, 1996; Lausch et al., 2013; Foster et al., 2018). On the mesenchymal aspect of the tooth, the biological interface between predentin/dentin is controlled by the odontoblasts and this dynamic interface will be maintained all life long (Linde, 1995; Orsini et al., 2007).

Although very thin, the PDL is vascularized and innervated. Innervation is an important parameter to prevent tooth ankylosis (Fujiyama et al., 2004). As mentioned above, the PDL is a soft tissue exposed to important loadings when mediating tooth anchoring to the mandibular bone. The bone matrix, as well as the cementum, is mineralized while the PDL ECM is not. In both cases, this is controlled by specific matrix constituents (i.e., BSP, osteopontin versus PLAP1/asporin), being present or not (Steitz et al., 2002; Hakki et al., 2006; Yamada et al., 2007; Foster et al., 2018) and associated growth factors (Dangaria et al., 2009). Asporin is considered a negative regulator of mineralization (Leong et al., 2012). At their insertion sites into either the alveolar bone or cementum, the PDL fibers are mineralized (McKee et al., 2013). *In vitro* re-mineralization approaches have been designed to investigate the mechanism involved in it. These experiments showed that the ECM itself retains the necessary information to reproduce the spatial specificity of soft-mineralized tissues boundaries (Lausch et al., 2013).

These biological interfaces allow relative movements while resisting biomechanical loads (Ho et al., 2007). Their structural heterogeneity makes it difficult to investigate, and require the development of appropriated technologies together with adjusted methodologies (Boskey, 1998; Shahar and Weiner, 2018 and citations therein). For a simultaneous visualization of collagen fibrils, crystals, and intracellular components, Shahar and Weiner (2018) strongly favor 3D-imaging: complementary methods have been developed for that purpose. They include high resolution confocal light microscopy (Costello et al., 2016), high resolution Micro-CT (Burghardt et al., 2011), serial block face SEM (Denk and Horstmann, 2004), focused ion beam SEM (Hirashima et al., 2020b), each method having its own limitations. Although on a different model, backscattered electron (Kerschnitzki et al., 2011), second-harmonic generation imaging (Campagnola et al., 2002) and synchrotron small angle X-ray scattering (Groetsch et al., 2019) have been used to visualize collagen fibers crossing the soft/hard tissue junction (Paris et al., 2017). Besides structural aspects, experimental approaches allowed addressing questions about functional points of view, such as the relationship between mechanical strain amplification, mineral deposition/resorption and the dynamic correlation between these processes (Grandfield et al., 2015). In physiological conditions, mineral apposition is much more slow and regular. The transformation of amorphous to crystalline phase of mineral might allow rapid local changes, but has not yet been documented in detail.

## Conclusion

Within the lower jaw of the mouse embryo, odontogenic and osteogenic condensations form almost simultaneously and next to each other. Although it has been published that mesenchymal cells have a natural tendency to aggregate, the two condensations remain separated by a loose interstitial mesenchyme. In opposition to the odontogenic and osteogenic ecto-mesenchymes originating from cranial NCDCs, cells from the interstitial mesenchyme do not express markers for neural crest origin. In all three zones, there is a progressive income of external cells: osteoclasts, cells mediating the vascularization or involved in the innervation, as well as stem/progenitor cells. Very soon all three mesenchymal areas show structural and physical differences. The secretion of distinct matrices will then support the different mineralization processes in odontogenic and osteogenic condensations. Besides its different origin, the interposed mesenchyme (prospective PDL) will not mineralize. This involves matrix-associated growth factors, some of which inhibit mineralization. These complementary processes lead to the formation of a functional complex: a tooth stabilized in a bone socket where movement still remains possible. While morphogenesis has been investigated in detail, mostly cellular aspects were taken into account in this review. Several questions remain, mainly concerning physical aspects within the three mesenchymes at early stages, such as the nature of differential cell-cell and cell-matrix interactions mediating cohesion as well as mechanosensing. Improving our knowledge about it would allow a better understanding of the mechanisms mediating/preventing the early mesenchymal cell segregation/compartmentalization and their condensation.

## Author Contributions

HL and RP contributed to the conception and writing of the manuscript. RP, ES, and EM revised it critically. ES and RP performed the illustrations. ES, RP, EM, and HL approved the content of the manuscript for publication and agreed to be accountable for all aspects of the work. All authors contributed to the article and approved the submitted version.

## Conflict of Interest

The authors declare that the research was conducted in the absence of any commercial or financial relationships that could be construed as a potential conflict of interest.
